# LATEST LASER AND LIGHT-BASED ADVANCES FOR ETHNIC SKIN REJUVENATION

**DOI:** 10.4103/0019-5154.41643

**Published:** 2008

**Authors:** Mohamed Lotfy Elsaie, Heather Woolery Lloyd

**Affiliations:** *From the Dermatology and Cutaneous Surgery Department, University of Miami Cosmetic Center, Fl, USA*

**Keywords:** *1064 nm laser*, *532 nm laser*, *ethnic skin*, *fractional photothermolysis*, *IPL*, *photoaging*

## Abstract

**Background:**

Advances in nonablative skin rejuvenation technologies have sparked a renewed interest in the cosmetic treatment of aging skin. More options exist now than ever before to reverse cutaneous changes caused by long-term exposure to sunlight. Although Caucasian skin is more prone to ultraviolet light injury, ethnic skin (typically classified as types IV to VI) also exhibits characteristic photoaging changes. Widespread belief that inevitable or irreversible textural changes or dyspigmentation occurs following laser- or light-based treatments, has been challenged in recent years by new classes of devices capable of protecting the epidermis from injury during treatment.

**Objective:**

The purpose of this article is to review recent clinical advances in the treatment of photoaging changes in ethnic skin. This article provides a basis for the classification of current advances in nonablative management of ethnic skin.

## Introduction

Advances in nonablative skin rejuvenation technologies have sparked a renewed interest in the cosmetic treatment of aging skin. More options exist now than ever before to reverse cutaneous changes caused by long-term exposure to sunlight. Although Caucasian skin is more prone to ultraviolet light injury, ethnic skin (typically classified as types IV to VI) also exhibits characteristic photoaging changes. Widespread belief that inevitable or irreversible textural changes or dyspigmentation occurs following laser or light-based treatments, has been challenged in recent years by new classes of devices capable of protecting the epidermis from injury during treatment. Ethnic skin represents the majority of the world's population and yet few research studies have targeted the safety and efficacy of cosmetic skin procedures in ethnic skin. This article highlights newer advances in nonablative ethnic skin rejuvenation and evaluates their safety and efficacy.

## Defining Ethnic Skin

In addition to grouping people of ethnic descent into classic Fitzpatrick categories of IV to VI to describe their propensity for sun reactivity, it is useful to describe how ethnic features and groups relate to one another. People of ethnic skin comprise the majority of the world's population. These include Asians, who can be subdivided into East Asians (Chinese, Japanese, Koreans), Southeast Asians (Indonesians, Malaysians, Singaporeans, Thais, Cambodians, Vietnamese), and South Asians (Bangladeshis, Indians, Pakistanis, Sri Lankans). Those from East Asia tend to have lighter skin color, although Koreans are generally more brown-skinned than the Chinese or Japanese. Southeast Asians have brown skin color while East Asians and Southeast Asians have a Mongoloid ethnic background. South Asians are of Caucasian ethnic background but have brown to dark brown skin.

## Photorejuvenation

Photorejuvenation is defined as the use of visible or infrared light energy sources to reverse the process of sun-induced or environmental damage to the skin.^1^ Visible disruption to the overlying epidermis should not occur while trying to accomplish this in a nonablative manner. The primary objective of nonablative rejuvenation is to improve aesthetic concerns characteristic of photoaged skin, including the appearance of dyspigmentation, static fine wrinkles, coarse texture, prominent pores, and telangiectasias. In contrast, chronological skin aging results in thin skin with reduced elasticity that retains normal skin pigmentation and texture.^2^ A secondary objective includes the recontouring of mild surface irregularities via subsequent dermal collagen remodeling.

In general, all races are susceptible to photoaging.^3^ However, it is clear that photoaging is delayed and less severe in patients with Fitzpatrick's skin phototypes IV to VI. This is due to the photoprotective role of melanin.^4^,^5^ Published studies on photoaging in black skin have been limited to African Americans. Photoaging is more prominent in lighter-complexioned African American individuals. In addition, photoaging may not be apparent until the fifth or sixth decade of life. Clinically, the features of photoaging in African Americans can include fine wrinkling, mottled pigmentation, and dermatosis papulosa nigra. African Americans also tend to manifest signs of skin laxity with aging. This is most evident in the nasolabial folds and jaws.^6^ Most studies on the treatment of photorejuvenation in ethnic skin utilize nonablative technologies that will be discussed in this paper.

### Fractional devices

Fractional photothermolysis (Fraxel SR, Reliant Lasers, Palo Alto, CA, USA) is a novel nonablative erbium:glass (1500 nm) laser treatment for facial rejuvenation.^7^ It is also used for the treatment of melasma and acneiform scarring.^8^ Fractional photothermolysis is performed with a midinfrared laser, which creates microscopic columns of thermal injury. These zones of thermal injury, termed microthermal zones (MTZs), have a diameter that is energy-dependent and ranges from 100 to 160 µm. The depth of penetration ranges from 300 to 700 µm at the energies commonly used for facial rejuvenation (8-12 mJ/MTZ).^9^ Relative epidermal and follicular structure sparing are responsible for rapid recovery without prolonged downtime. Melanin is not at risk of selective, targeted destruction; therefore, fractional resurfacing has been used successfully in patients with skin of color. Kono *et al.*^10^ have described the use of the Fraxel in 35 type IV and V Asian patients and concluded that increased density was more likely to produce swelling, redness, and hyperpigmentation when compared to increased energy. In this study, the authors concluded that patient satisfaction is significantly higher when their skin is treated with high fluences than when treated with high densities. They concluded that fractional photorejuvenation can be safe and effective in darker ethnic skin types.^10^

Prior studies using fractional photothermolysis have demonstrated its effectiveness in the treatment of photodamaged skin; however, only preliminary results have been reported regarding its use for scars. Given the rapid healing associated with this procedure and its known effect on collagen remodeling, this study was designed to prospectively evaluate the use of fractional photothermolysis in the treatment of atrophic scars. Fiftythree patients (skin phototypes I-V) with mild-to-moderate atrophic facial acne scars received monthly treatment with a 1550 nm erbium-doped fiber laser (Fraxel, Reliant Technologies Inc., San Diego, CA). Clinical response to the treatment was determined by two independent assessors at each treatment visit and six months after the final treatment session, by using a quartile grading scale. Side effects and patient satisfaction were monitored at each follow-up visit. Ninety-one per cent of the patients had at least 25-50% improvement after a single treatment, whereas 87% of the patients receiving three treatments demonstrated at least 51-75% improvement in the appearance of their scars. Moreover, age, sex, and skin phototype also did not significantly affect the observed clinical responses. Hence, it was concluded that fraxel procedures were effective in acne scar treatment for skin of color.^11^

### 532 nm Laser

Although not a prominent feature of ethnic skin, treatment of the pigmented and telangiectatic component of photoaging has been reported in ethnic skin.^12^,^13^ Rashid and colleagues reported the use of a quasicontinuous wave 532 nm laser in the treatment of lentigines in type IV skin patients.^13^ They showed 50% improvement in lesion clearance, with a 10% incidence of hyperpigmentation and 25% incidence of hypopigmentation after multiple treatments. These side effects abated after two to six months. Lee reported 150 patients with skin types I to V who were treated in multiple sessions with 532 nm (4 mm spot, 6-15 J/cm^2^, 30-50 millisecond pulse duration), 1064 nm (10 mm spot, 24-30 J/cm^2^, 30-65 millisecond pulse duration), or a combination of both.^11^ Sapphire-tipped contact cooling was utilized. Improvement in erythema, texture, pigmentation, and rhytids was reported in both study arms but was highest in the combination group. An incidence of 5% postinflammatory hyperpigmentation was reported in patients with types III and IV skin treated with the 532 nm laser alone, which resolved after 4-6 weeks.^12^ The use of conservative settings to achieve the desired results is prudent. Following these guidelines, the clinician is most likely to achieve a favorable result with the least unwanted side effects. Test spots are necessary to assess the initial patient response and decrease the risk of hypopigmentation, which is often very difficult to treat.

### 1064 nm Laser

Long-pulsed and Q-switched 1064 nm lasers target melanin as well as hemoglobin and water. Although safer for darker skin, there is a diffuse heating of dermal tissue owing to the deep, penetrating nature of 1064 nm with a typical dispersion depth of 5-10 mm.^1^ One study has shown evidence of improvement with a Q-switched 1064 nm laser for nonablative treatment in type IV skin.^13^ Sun-damaged 4 cm × 4 cm areas of infraauricular skin were exposed to a 1064 nm Q-switched Nd:YAG laser at a fluence of 7 J/cm^2^ and a 3 mm spot size. Two laser passes with a 10-20% overlap, were used on all subjects in an attempt to promote petechiae as the visible end point. Petrolatum dressings were applied for a week after treatment. Three millimeter punch biopsy specimens were taken from each subject before treatment. Photographs were taken of the biopsy sites. Three months after the last treatment, another biopsy specimen was taken from a different previously treated area. Histological specimens were evaluated blindly by a board-certified dermatopathologist. Four out of six skin biopsy specimens obtained three months after the last laser treatment, showed mild fibrosis with histological improvement in pretreatment solar elastosis. There was a mildly thickened, upper papillary collagen zone, with an improvement in the organization of collagen fibrils. The remaining two specimens showed no changes. Clinically, none of the treated, nonbiopsied areas showed any evidence of pigmentary changes or scarring.^13^

Another study utilized the 1064nm Nd:YAG (Laser genesis) for the rejuvenation of facial skin of types I-V. Patients' and masked physician assessment demonstrated overall improvement. Specific improvement was also demonstrated in coarse wrinkles and skin laxity. No adverse events were noted in this study.^14^ Studies have all confirmed the effectiveness and low risk of complication associated with Nd:YAG for rejuvenation in skin of color.

### Intense Pulsed Light

Another device for photorejuvenation of ethnic skin is Intense Pulsed Light (IPL). IPL is produced by a noncoherent flashlamp-pumped light source that is capable of emitting light from 500 to 1200 nm.^15^ The use of cutoff filters allows the elimination of some of the shorter wavelengths of the visible light spectrum to limit melanin absorption. Different pulse widths can be chosen so that appropriate parameters match the thermal relaxation times of the targets.^16^ Cooling of the epidermis is achieved with contact cooling in the device head or with external cooling devices.

Negishi and colleagues were among the first to investigate the use of IPL in types IV and V Japanese patients using pulsed light devices. They applied a thin layer of ice-cold gel and they utilized a 550 nm cutoff filter. Settings were 28 to 32 J/cm^2^ and 2.5-4.0 and 4.0-5.0 millisecond pulse durations. Excellent results were reported in 73 out of 97 patients.^17^,^18^ No evidence of dyspigmentation was reported in either series. Negishi and colleagues have also employed UV photography to identify and treat subclinical epidermal hyperpigmentation with IPL in skin of color.^19^ Although IPL has been utilized with success in skin of color, treatment of such patients should utilize conservative settings to achieve a favorable result with the least unwanted side effects.

### Light-emitting diode (LED)

Apart from the light- and laser-based devices described above, three newly described nonablative technologies have been used for treatment in ethnic skin types. Light Emitting Diodes (LEDs) represent the latest advancement in visible spectrum, monochromatic light therapy for photoaged skin. Typically, LEDs in devices are arrayed in panels, and each emits visible light in a ±10-20 nm band around the dominant emitted wavelength. Energy output is less than 25 W, representing a fluence of about 0.1 J/cm^2^. The Gentlewaves LED device (Light Biosciences, Virginia Beach, VA, USA) recently received approval from the US Food and Drug Administration for the treatment of periorbital wrinkling.^20^ In brief, this device is thought to act by targeted stimulation of fibroblastic mitochondrial metabolic activity, concomitant upregulation of procollagen, and downregulation of matrix metalloproteinase I.^21^,^22^

### Radiofrequency (RF)

Radiofrequency (RF) is an electromagnetic radiation in the frequency range of 3 kHz to 300 GHz. The primary effects of RF energy on living tissue are considered to be thermal. The main goal of these new frequency-based devices is to heat specific layers of the skin. Directed use of RF can induce dermal heating and cause collagen degeneration. Wound healing mechanisms promote the remodeling of collagen and wound contraction, which ultimately enhances the appearance of mild to moderate skin laxity. Preliminary studies with one device (Thermacool, Thermage Inc, Hayward, CA, USA) have reported efficacy in the treatment of laxity involving the periorbital area and jowls.^23^ As RF energy is not dependent on specific chromophore interaction, epidermal melanin is not at risk of destruction and treatment of all skin types is possible.

Kushikata *et al.*^24^ reported the use of RF in a series of 85 Asian patients of dark skin types and concluded that RF treatment was very satisfactory for skin tightening in Asian facial skin. RF appears to be a promising means of photorejuvenation in ethnic skin.

### Infrared Tightening

Improvement of facial and cervical skin laxity has been difficult to achieve without surgical procedures. A device called the Titan (Cutera, Inc., Brisbane, California) uses infrared (IR) light to volumetrically heat the dermis. It is designed to thermally induce collagen contraction with subsequent collagen remodeling and neocollagen synthesis. The epidermis is protected via pre-, parallel, and posttreatment cooling. No anesthesia is necessary as there is minimal to no discomfort during the procedure. Improvements in skin laxity and facial and neck contours have been achieved with this device, although results can vary. This variation may be caused by patient variability and differences in technique.^25^

Few studies have addressed the efficacy and safety of infrared use in darker skin, however Chua *et al.* investigated the use of IR on 21 patients of Fitzpatrick skin types IV and V. Eighty-six per cent of the patients had improvement as measured by the physician's assessment at their six months' follow-up visit. Hence, Chau *et al.* concluded that direct application of infrared light with epidermal cooling is effective in achieving gradual, mild-to-moderate clinical improvement in the treatment of facial and neck skin laxity. The procedure is associated with minimal downtime and is safe for use in darker skin types IV and V.^26^

### Plasma skin regeneration (PSR) technology

Plasma is the fourth state of matter acquired by ionizing a gas. One example of this is the light we see with lightning. The electricity (energy) discharged from the clouds to the earth heats up the air (gas) and converts it into plasma.^27^ A basic understanding of skin structure is required to understand how PSR works. Briefly, skin consists of three layers: the epidermis (uppermost layer), dermis (middle layer) and subcutis (lower fat layer). The epidermis contains pigment-producing cells called melanocytes, which are responsible for skin coloring. The dermis is made up of collagen and elastin fibres that provide skin with strength, toughness, elasticity and pliability. The appearance and characteristics of skin change as the body ages. The epidermis becomes thinner so that blemishes become more visible, and collagen in the dermis is gradually lost, which contributes to the formation of facial lines, sagging skin and wrinkles.^28^

To date, there are five anti-aging treatment regimens - PSR 1, PSR 2, PSR 3, PSR 2/3 combination and a fifth newly FDA approved one. A particular regimen is chosen according to the severity of the problem being treated and the recovery time available. The fifth treatment is a new FDA-approved, anti-aging procedure for treating nonfacial areas of the body. All protocols could be used for lines; however, higher energy treatments are needed for skin tightening. Studies have shown that the thermal energy at 1.0 and 2.0 J was limited to the epidermis and dermoepidermal junction. At 3.0 and 4.0 J, the thermal injury reached the papillary dermis. PSR 1 protocol uses a low-energy treatment spaced three weeks apart. PSR 2 uses a single high pass 3.0-4.0 J energy treatment with a recovery time of 5-7 days. PSR 3 uses two high-energy passes (3.0-4.0 J) with a recovery period of 6-10 days. A fourth protocol uses a combination of PSR 2 and PSR 3 and the fifth one uses very low energy (0.5 J) in a series of three treatments at three-week intervals.^29^ Few studies have been carried out on subjects so far; however, Kilmer,^30^ Poter,^31^ Benstein^32^ and Bogle^33^ have all demonstrated the low risk and efficacy of using such technology in all skin types.

### Summary

In conclusion, laser procedures in darker skinned patients are challenging but can be successfully achieved if certain treatment guidelines are followed. Discussion of risks and patients' expectations are essential in treating the darkerskinned patient population. Pre- and postlaser cooling can be helpful to minimize side effects and improve patients' comfort. This is especially true with laser hair removal. Photorejuvenation can be successfully achieved with low risk when appropriate settings are used. Fractional technology has increased treatment options for rhytides and atrophic scars. Although there are few studies on LED treatments in skin of color, this type of treatment can be used either as a primary or adjunctive treatment modality with apparently low risk. The 532 nm laser has proved to be risky in skin of color and conservative guidelines should be followed when using it. On the other hand, the 1064 nm laser may offer greater safety when treating ethnic skin albeit still risky in type VI skin. The IPL is a safe option for treating skin of color although it is advisable to limit its use for skin types V and VI. Finally, radiofrequency and newer tightening technologies are safe and highly reliable to use for ethnic skin. However, it is prudent to use conservative settings to achieve the desired results when treating darker skinned patients. The clinician is most likely to achieve a favorable result with the least unwanted side effects if these guidelines are followed.
